# High-throughput techniques enable advances in the roles of DNA and RNA secondary structures in transcriptional and post-transcriptional gene regulation

**DOI:** 10.1186/s13059-022-02727-6

**Published:** 2022-07-18

**Authors:** Ilias Georgakopoulos-Soares, Candace S. Y. Chan, Nadav Ahituv, Martin Hemberg

**Affiliations:** 1 Department of Bioengineering and Therapeutic Sciences, University of California San Francisco, San Francisco, California USA; 2 Institute for Human Genetics, University of California San Francisco, San Francisco, California USA; 3 Evergrande Center for Immunologic Diseases, Harvard Medical School and Brigham and Women’s Hospital, Boston, MA USA

## Abstract

The most stable structure of DNA is the canonical right-handed double helix termed B DNA. However, certain environments and sequence motifs favor alternative conformations, termed non-canonical secondary structures. The roles of DNA and RNA secondary structures in transcriptional regulation remain incompletely understood. However, advances in high-throughput assays have enabled genome wide characterization of some secondary structures. Here, we describe their regulatory functions in promoters and 3’UTRs, providing insights into key mechanisms through which they regulate gene expression. We discuss their implication in human disease, and how advances in molecular technologies and emerging high-throughput experimental methods could provide additional insights.

**Supplementary Information:**

The online version contains supplementary material available at 10.1186/s13059-022-02727-6.

## Introduction

**Fig. 1:**
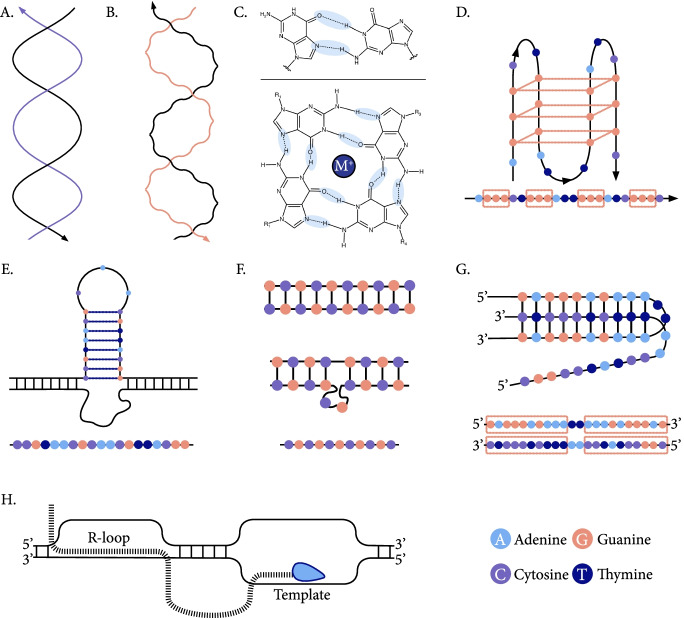
Schematic overview of Non-B DNA enrichment relative to gene features. Higher density of non-B DNA structures is observed at promoter regions, 5’UTRs, regions flanking splice sites and at the 3’UTR. Formation of secondary structures is also facilitated by negative supercoiling and at actively transcribed regions relative to the direction of the transcribing RNA polymerase [2, 3]

The canonical conformation of the DNA molecule is a double helix which under physiological conditions forms a stable structure known as B DNA. However, certain environments and sequence motifs favor other DNA conformations, known as non-canonical secondary structures. Moreover, many of the structures are also present in RNA molecules. These structures include perfect and imperfect hairpins, cruciforms, slipped structures, R-loops, G-quadruplexes, i-motifs, Z-DNA, Z-RNA, triple-stranded DNA, RNA and hybrid structures. Sequences that are predisposed to secondary structure formation are enriched at regulatory regions, including open chromatin regions, promoters, 5’UTRs and 3’UTRs [1, 2] (Fig. 1). In particular, they are over-represented and positioned relative to key gene features, such as transcription start and transcription end sites, splice junctions and translation initiation regions, while their formation is associated with transcriptionally active loci [4–11]. Thus, secondary structures can have a functional impact since promoter regions control transcription initiation, while 3’UTRs have a number of functions, including impacting the stability of the transcript and its rate of degradation, providing binding sites for regulatory elements such as miRNAs and RNA binding proteins (RBPs), and containing signals for the localisation of the transcript in the cell.

Non-canonical secondary structures often impact gene expression at the DNA and RNA levels, having key roles in gene regulation. Nevertheless, the role of DNA and RNA secondary structures in transcriptional and post-transcriptional regulation remains incompletely understood. Incorporating these effects in models of gene regulation has the potential to improve our understanding of how cells fine-tune gene expression and enable the development of novel therapies. Thanks to technological advances and novel computational and experimental methods several powerful tools have recently become available that have the potential to fundamentally alter our understanding of the role of secondary structures in gene regulation. At the same time, DNA and RNA secondary structure formation has been related to a number of diseases and are emerging as novel therapeutic targets [12–16]. In this review, we summarize recent advances that are changing our understanding regarding the roles of DNA and RNA secondary structures in promoters and 3’UTRs with a particular focus on transcriptional and post-transcriptional gene regulation and discuss emerging prospects, challenges and therapeutic opportunities.

## Non-canonical DNA and RNA secondary structures

**Fig. 2:**
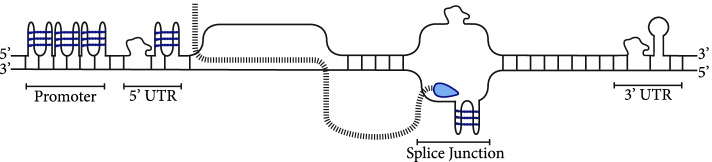
Schematics of DNA and RNA structures. **A** The canonical right handed double helix, also known as B DNA secondary structure. **B** Z-DNA forms a left-handed double helix. **C** G-quadruplexes are formed by the stacking of multiple G-quartets held together by Hoogsteen hydrogen bonds (top). Four guanines establish hydrogen bonds with each other to form a G-quartet (bottom). Hoogsteen hydrogen bonds are highlighted in blue. The monovalent cation that can stabilize the G-quadruplex structure is marked with M. **D** Hairpins are formed at inverted repeats, in which the stem base pairs hybridize with hydrogen bonds, while the loop remains single-stranded. **E** Slipped-strand mispairing at tandem repeats results in slipped structure formation. **F** Depiction of a homopurine-homopyrimidine sequence with mirror symmetry. H-DNA is a triple helix secondary structure where the third strand hybridizes with Hoogsteen hydrogen bonds with the duplex DNA, while the fourth strand remains single stranded. **G** R-loops are formed co-transcriptionally at the template strand. The nascent RNA produced by the RNA-polymerase hybridizes with the template strand to form an R-loop structure, while the non-template strand remains single-stranded

The characterization of the DNA secondary structure in a landmark paper [17] provided fundamental insights into how genetic information is stored and used. Double stranded DNA is composed of two helices held together by hydrogen bonds, and most often adopts the canonical right-handed double helical secondary structure, also known as B-DNA (Fig. 2a), with 10.5 residues per turn [18]. Similarly to B-DNA, A-DNA is also right-handed and double helical albeit with 11 residues per turn and forms more readily at GC-rich regions [19, 20]. Larger distortions in the DNA structure occur at sequences that are predisposed to alternative conformations and are collectively termed non-B DNA, encompassing multifarious DNA secondary structures [21]. Although several DNA and RNA secondary structures are shared, the two molecules sometimes substantially differ in their thermodynamic stability and likelihood of secondary structure formation. The single strandedness of RNA molecules enables long range base pairing interactions, while physical constraints in the DNA molecule restrict such interactions to directly adjacent sequences. Below we describe the DNA and RNA secondary structures that are discussed in this review.

Z-DNA is a left-handed double helical structure (Fig. 2b) that is formed primarily at regions with alternating purine pyrimidine tracts, particularly at GC repeats [22–24]. Z-DNA is less energetically favorable than B DNA under physiological conditions, and consequently it requires negative supercoiling. Z-DNA formation has been associated with active transcription [25], supporting the correlation between Z-DNA levels and transcription observed in the past [26].

G-quadruplexes are nucleic acid structures held together with Hoogsteen hydrogen bonds between guanines that form stacked G-tetrads (Fig. 2c). Hoogsteen base pairing refers to non-Watson–Crick base pairing as shown in Fig. 2c. These bonds connect four guanines forming a square planar arrangement which is known as a G-quartet. Stacking multiple G-quartets results in the formation of a G-quadruplex. G-quadruplex formation is driven by the inherent propensity of guanines to self-assemble in the presence of monovalent cations into planar structures [27]. Traditionally, they are classified as parallel, antiparallel or hybrid depending on the folding topology [28]. However, recent work has shown the existence of additional, more complex arrangements [29–31].

DNA and RNA hairpins can form at perfect and imperfect inverted repeats. Inverted repeats are composed of two adjacent copies of the same sequence, one of which is found in the reverse complement orientation (Fig. 2d). A hairpin is held together by hydrogen bonds between the two complementary arms, while the spacer remains single stranded. A closely related structure is cruciforms, consisting of two hairpins and a 4-way junction [32]. It has been shown that sequence properties of inverted repeats, including spacer and arm length, interruptions and nucleotide composition can affect the folding kinetics and stability of hairpins and cruciforms. Inverted repeats with an arm length of >6 nts have been shown to form *in vivo* in *Saccharomyces cerevisiae* [33]. Hairpin formation dynamics have been studied in detail by varying the spacer lengths, arm lengths, and nucleotide composition, as well as by examining the folding and mutagenic potential [33–36]. For instance, arms with higher GC content display more stable hairpin formation [36].

Slipped structures form at consecutive repeat sequences, in which one repeat unit misaligns with the second repeat unit on the opposite strand [37] (Fig. 2e). Direct and short tandem repeats can form these structures, and due to inefficient repair they are often expanded or contracted [38]. There are over one million tandem repeats in the human genome, and many are polymorphic [16].

Intramolecular triple stranded DNA (H-DNA) forms at homopurine-homopyrimidine stretches that contain a mirror symmetry [39, 40]. One strand folds back, joins the double-stranded DNA, and is held with Hoogsteen or reverse-Hoogsteen bonds. The other strand remains single, and the result is a triple helical structure (Fig. 2f). Intermolecular triplexes can occur between DNA molecules, RNA molecules, or as a hybrid involving a DNA and a RNA molecule [41].

During transcription, dynamic hybrid structures between DNA and nascent RNA transcripts can be formed [42, 43]. One example is R-loops, where an RNA molecule invades and pairs up with one DNA strand, while displacing the other (Fig. 2g). Formation and stabilization of R loops is particularly favorable when the non-template strand is G-rich, and it can also be promoted by DNA supercoiling, the presence of DNA nicks, and the formation of G-quartets [44, 45]. The continuous activity of DNA/RNA helicases and ribonucleases H (RNAse H1 and H2) maintain R-loop formation at low levels [45]. Interestingly, R-loops and G-quadruplexes were both found to be unwound by the helicase DHX9 [46]. This helicase activity is important to avoid single-stranded DNA damage and to preserve genomic stability. It has been shown that R-loops can occupy ~5% of the human genome, and that they are depleted in intergenic regions relative to genic regions. However, R-loops are highly dynamic and it has been estimated that <10% of loops are formed at any point in time [47, 48]. Formation of R-loops shows a remarkable strand asymmetry, with more than 90% of R-loops occurring co-linearly with transcription [48].

## High-throughput techniques to identify non-B DNA structures

A number of biophysical and biochemical methods as well as highly sensitive and specific assays have been developed to study the formation of DNA and RNA structures *in vitro* [49]. However, *in vivo* identification of secondary structures, even in cell cultures, has remained more challenging. Recently, permanganate footprinting was combined with genome-wide sequencing to identify transiently formed single stranded DNA regions [2], providing a readout for genome-wide non-B DNA formation. Furthermore, antibodies with high affinity and specificity have been developed targeting specific secondary structures and enabling their visualization [50–53], while other methods use the cleavage of DNA-RNA hybrids by specific nucleases to map secondary structures or nucleotide hybridization within RNA molecules to determine their folding [2, 54, 55].

Genome-wide maps of sequences that can form G-quadruplexes *in vitro* under favorable conditions have been generated using a modified sequencing method that stalls at G-quadruplexes [29, 56]. This technique has been termed G4-seq. Similarly, rG4-seq, which is a variant of G4-seq, has enabled transcriptome-wide identification of RNA G-quadruplexes [57]. Recently, a novel method called G4-miner was used for genome-wide profiling of G-quadruplexes from standard whole-genome sequencing based on deviations in sequencing quality, with drop in sequencing quality at G-quadruplex structures [58]. Antibodies with high affinity for G-quadruplexes have been used for G4 ChIP-seq experiments to demonstrate genome-wide G-quadruplex structure formation in human cells [1, 59]. Crucially, the number of loci discovered forming G-quadruplexes by ChIP-seq *in vivo* is ~10,000 while G4-seq *in vitro* identifies ~700,000 peaks [29, 56, 59]. This discrepancy could be the result of rapid resolution of G-quadruplexes by helicases in the cellular environment [60], it could reflect differences in the genomic locality, associated with epigenetic changes and chromatin accessibility [61] and could be biased by the chemical perturbation with K^+^ or Pyridostatin (PDS) in G4-seq experiments. In addition, in ChIP-seq this discrepancy could also be explained by the antibody being able to recognize only certain G-quadruplexes. Antibody-associated differences are indeed observed between BG4 and D1 antibodies [1, 62]. Large variability in the number of peaks has also been observed in G4-seq experiments where the G-quadruplex stabilization method seems to influence the results with substantial differences in PDS and K^+^ treatments. PDS is a small molecule compound that has been shown to bind to G-quadruplexes, leading to their stabilization [63]. Similarly, K^+^ ions interact with G-quadruplexes and stabilize them. High-throughput G-quadruplex detection methods identify sites that do not conform to the consensus G-quadruplex motif. However, these high-throughput methods entail certain limitations. These include the inability to discern the G-quadruplex forming potential between different cell types or to measure temporal effects; they do not provide information about the kinetics and thermal stability of G-quadruplexes at individual loci. More recently, single molecule G-quadruplex tracking in living cells, using a G-quadruplex specific fluorescent probe, has indicated dynamic fluctuations between folded and unfolded states [64].

Z-DNA binding proteins have a plethora of biological functions, and several proteins that have a Z-DNA binding domain (Zα) have been identified [65–67]. Isolation of proteins that bind preferentially to Z-DNA over B-DNA identified ADAR1 [68] and the specificity has been used in ChIP-seq experiments to identify sites of Z-DNA formation in the human genome [68]. Methods have also been developed to identify DNA:RNA structures from cells such as isolation of DNA-associated RNA coupled with high-throughput sequencing [69]. *In vitro* R-loop identification was initially potentiated through the development of an antibody with high specificity to its secondary structure [50, 70, 71]. Advances in molecular technologies have resulted in numerous variants of the S9.6 RNA:DNA hybrid antibody, and by combining them with nucleases that cleave DNA:RNA hybrids systematic studies of R-loops have been made possible [70, 71]. R-ChIP was the first method that mapped genome-wide R-loops using catalytically dead RNase H coupled with by amplification of immunoprecipitated DNA [72]. The development of DNA-RNA immunoprecipitation sequencing (DRIP-seq) has further enabled genome-wide profiling of R-loops and the identification of genomic sites that form R-loops with higher propensity [70, 73], while recent antibody-independent nuclease-based methods, include MapR and BisMapR, which provide high-resolution genome-wide detection of R-loops [55, 74].

## Secondary structures are involved in transcriptional regulation at promoters

**Fig. 3:**
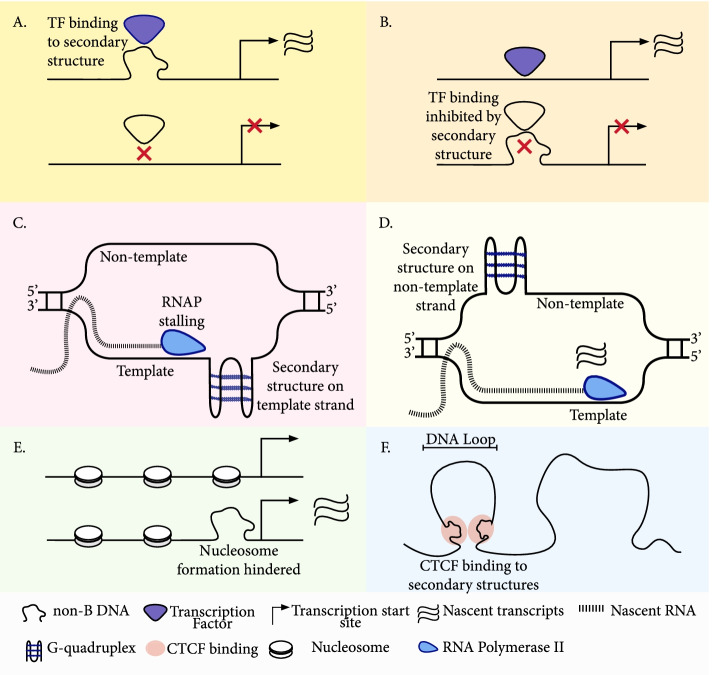
Functional roles of secondary structures in promoter regulation. **a** Non-B DNA structure formation can mediate transcription factor binding and promote transcription [81–86]. **b** Non-B DNA structure formation can hinder transcription factor binding to inhibit transcription [87, 88]. **c** RNAP stalling at the G-quadruplex structure formed at the template strand [81, 89]. **d** G-quadruplex structure formation at the non-template strand enables the template strand to remain single-stranded and promotes expression [90]. **e** Non-B DNA structure formation can hinder nucleosome formation to promote transcription [1, 91–93]. **f** Non-B DNA structures can promote long range DNA looping [94–98]

Non-canonical DNA secondary structures at the promoter play an important role across a range of processes. First, DNA secondary structures can act as landing pads for certain transcription factors [25, 75–80], many of which show preferential binding relative to B DNA (Fig. 3a, b). Second, the formation of non-canonical DNA secondary structures can act as a physical barrier for nucleosome formation [48, 91, 99, 100], thereby promoting accessibility (Fig. 3c). Third, secondary structures may influence genome organization and long-range DNA looping [94–97] (Fig. 3d). Fourth, regions with high propensity of forming secondary structures are associated with RNAPII pausing [72, 81, 89], a phenomenon important for several regulatory processes including promoter-proximal pausing, exon recognition, splicing and transcription termination (Fig. 3e, f).

Arguably the most studied non-canonical DNA secondary structure in promoter regions is the G-quadruplex. Estimates using the consensus G-quadruplex motif show that >50% of extended promoter regions harbor at least one G-quadruplex motif [101]. The orientation of G-quadruplexes relative to transcription direction is skewed with a higher frequency at the template strand upstream of the TSS and at the non-template strand downstream [102, 103]. In addition, TATA-less promoters have a substantially higher G-quadruplex density, both near the transcription start site and in the broader promoter region [104]. Moreover, genome-wide G4-seq experiments have demonstrated that a large proportion of G-quadruplexes do not adhere to the consensus motif, with inter-molecular G-quadruplexes as well as structures with bulges, disruptions and longer loops being over-represented in promoter regions [29]. The enrichment of G-quadruplexes in promoters relative to other sites in the genome was also recapitulated in G4 ChIP-seq experiments. Again, it was found that only 21% of G-quadruplex peaks contain a consensus motif [1], indicating a high G-quadruplex sequence diversity.

Additionally, epigenetic modifications influence the likelihood of G-quadruplex formation and stability; open chromatin regions and highly transcribed genes are enriched for G-quadruplex structures [1, 105], while cytosine methylation of G-quadruplexes increases stability [106, 107]. Conversely, G-quadruplexes are enriched at CpG sites and their formation is associated with CpG island hypomethylation through inhibition of DNA methyltransferase 1 enzymatic activity [108]. The epigenetic guanine conversion to 8-oxo-7,8-dihydroguanine through DNA damage has also been associated with increased G-quadruplex formation in promoters [109]. Topologically associated domains (TADs) represent self-interacting genomic sites, and G-quadruplexes are enriched at TAD boundaries [96, 97] where they interact with both CTCF [97] and Yin and Yang 1 (YY1) [95] to facilitate long-range DNA looping (Fig. 3f). In particular, YY1 binds directly to the G-quadruplex structure and stabilization of the G-quadruplexes results in coordination of the genes in the same DNA loop [95].

The relationship between G-quadruplex formation at promoters and gene expression levels varies, and likely depends on several factors, including strand orientation, positioning relative to TSS, biophysical properties of the G-quadruplex, presence of transcription factor binding sites in the vicinity of the G-quadruplex, and epigenetic marks. Thus, it has been shown that G-quadruplexes can both promote [110–112] and inhibit expression [4, 113]. However, since G-quadruplexes are landing pads for a number of proteins (Fig. 3b, c), e.g. SP1 [77], NM23-H2, CNBP25 and Nucleolin [114], it is difficult to estimate their contribution towards gene expression [115, 116]. Indeed, multiple transcription factor binding sites have been found to coincide with G-quadruplexes significantly more often than expected by chance. A recent high-throughput massively parallel reporter assay (MPRA) study examined the contribution of promoter G-quadruplexes and reported a positive correlation between their presence and expression levels. However, after accounting for GC-content differences, G-quadruplexes were either not significantly associated with expression levels or were associated with reduced gene expression [117]. The study also identified a bias with G-quadruplexes at the template strand resulting in lower expression levels. These results suggest that nucleotide composition is a major confounder in understanding the contribution of G-quadruplexes to gene expression.

Z-DNA sequences are enriched in promoters upstream of the TSS [118, 119], where they can act as nucleosome boundary elements to promote open chromatin [91, 120] (Fig. 3c). However, there is conflicting evidence regarding their impact on gene expression levels. It has been reported that Z-DNA forming in the first exon of *ADAM12* acts as a repressor [8, 121]. By contrast, studies in yeast have shown that Z-DNA can serve as activators [122]. Similarly, it has been found that formation of Z-DNA at the *CSF1* gene stabilizes the open chromatin and aids the recruitment of RNA polymerase [123], while in the promoter region of *HO-1* formation of Z-DNA precedes the recruitment of RNAPII, resulting in transcriptional activation [124]. The latter examples are supported by a recent MPRA where Z-DNA was shown to be a positive regulator of gene expression [117].

R-loops are enriched at promoters [70], with a 2-fold enrichment over background levels [48], and transcription is positively correlated with their frequency at promoter regions [125, 126]. R-loops can be stabilized at G-quadruplex sites [127] and R-loop formation is more frequent at CpG-island containing promoters where they increase expression through reduced methylation levels [70]. Formation of R-loops in promoters can facilitate histone modifications and is associated with open chromatin regions and GC-skew [43, 48, 128, 129]. The DNA-RNA helicase Senataxin [130], the RNA helicase DHX9 [131], the RNases H1 and H2 [132, 133] and topoisomerases inhibit the formation or cleave R-loops, while their accumulation can cause DNA damage, genome instability and is deleterious [45]. Roles for R-loops in long non-coding RNA (lncRNA) transcription have also been shown, in which R-loop formation can induce lncRNA generation which in turn act as transcriptional inducers [134, 135]. Specifically, at promoters R-loop formation can generate antisense lncRNAs, and their removal results in the inhibition of antisense lncRNAs [135]. R-loops have been found to be important for lncRNA function, for example the HOTTIP lncRNA recruits CTCF and cohesin, interacts with R-loop associated proteins and induces the formation of R-loops at TAD boundaries, which in turn regulates and reinforces those boundaries [98]. In *Arabidopsis thaliana*, the lncRNA APOLO recognizes distal targets via R-loops to epigenetically silence them [136]. There is also accumulating evidence for roles of R-loops in promoter-proximal RNAPII pausing [72, 137–139]. Transcription perturbation experiments have provided evidence for a strong link between R-loop induction and RNAPII pausing near transcription start sites [72]. Another line of evidence comes from a study which showed that BRCA1 can resolve R-loops at promoter-proximal RNAPII pausing sites [139], while in BRCA1 mutant cells, R-loops accumulate at the 5′ end of genes resulting in promoter-proximal RNAPII pausing [139].

Triple helix structures (single-strand RNA hybridizing to double-strand DNA with Hoogsteen bonds) have been shown to contribute towards genome organization [94], and some of the most thoroughly studied lncRNAs, e.g. HOTAIR, MEG3 and PARTICLE, form triple stranded DNA-RNA hybrids to perform epigenetic modifications and to regulate gene expression [140–142]. Short tandem repeats are enriched in promoters [143, 144] and enhancers [145] and have multiple regulatory roles, e.g. enabling the formation of slipped structures, G-quadruplexes and R-loops, creating additional or destroying existing transcription factor binding sites [146–150], altering DNA methylation [151, 152], and influencing nucleosome positioning [153]. Polymorphic short tandem repeats are estimated to account for 10-15% of the variance in gene expression [146]. In particular, 10-20% of eukaryotic genes and promoters contain an unstable repeat tract [154], and changes in repeat length at short tandem repeats are causal expression quantitative trait loci (eQTLs) [155]. In yeast, up to a quarter of promoters contain a highly variable tandem repeat sequence that affects gene expression [156]. Moreover, a study of 17 human tissues identified ~28,000 short tandem repeats for which the number of repeat units was associated with the expression of nearby genes [78].

## Secondary structures at the 3’UTR in transcriptional and post-transcriptional regulation

The stability of an mRNA transcript and its rate of degradation are major contributors to expression levels. Perhaps the most important determinant of mRNA half-life is the 3’UTR which contains polyadenylation signals, binding sites for RBPs, and miRNA target sites. Multiple alternative polyadenylation signals are found in the majority of 3’UTRs [157], which can alter the length of the 3’UTR, influencing mRNA structure, stability and translation efficiency [158]. Moreover, the folding of the 3’UTR can influence the maturation, localization and metabolism of the transcript [159–161]. Although highly structured mRNA molecules are less stable [159], studying the role of RNA structure is complicated by the fact that 3’UTR regions in humans are more structured *in vivo* than *in vitro* [162].

**Fig. 4:**
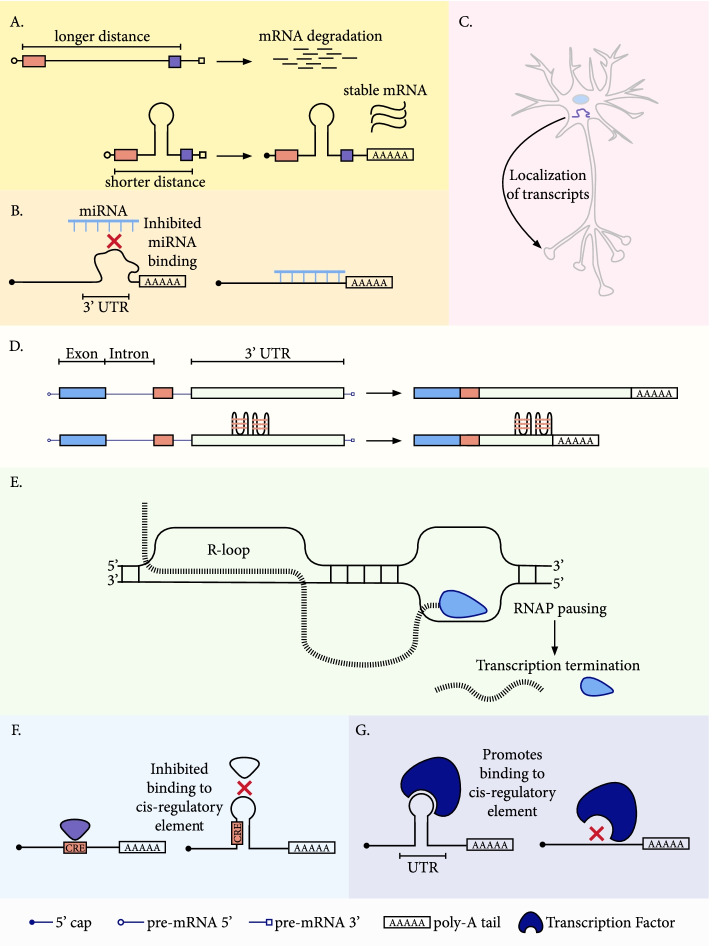
Functional roles of secondary structures in transcription termination. **a** Juxtaposition of the poly(A) signal and the polyadenylation sequence [160, 163]. Formation of a hairpin structure brings the poly(A) signal in closer proximity to the polyadenylation sequence, resulting in higher expression levels. **b** Modulation of miRNA binding [164–166]. **c** Formation of the secondary structure enables transcript localization [161], **d.** Modulation of alternative polyadenylation by structure formation [167]. **e** R-loop formation at the 3’UTR promotes transcription termination. **f** Concealment of cis-regulatory elements [168, 169], **g** Exposure of cis-regulatory elements [170–174]

Secondary structure formation within the 3’UTR impacts expression levels in a variety of ways. For instance, although longer distance between polyadenylation signals and polyadenylation [poly(A)] sites is associated with a shorter RNA half life, secondary structures may juxtapose the poly(A) signals and the polyadenylation sequences [160] (Fig. 4a). The formation of hairpin structures impacts mRNA stability distinctly from the AU-rich sequences [168, 175]. A recent MPRA study quantified the contribution of secondary structures found in the 3’UTR, demonstrating their contribution alongside RBPs and miRNAs [176].

G-quadruplexes have a 2-fold enrichment in human 3’UTRs, and they are 1.28-fold more frequent at the template than the non-template strand [167, 177]. Overall ~15% of these 3’UTRs harbor at least one G-quadruplex motif, but the enrichment is ~30% for neuronal mRNAs that locate to dendrites. For instance, G-quadruplexes at the 3’UTR of two post-synaptic proteins, PSD-95 and CaMKIIa, are necessary and sufficient for their localization in dendrites [161] (Fig. 4b). Studies have also found roles for G-quadruplexes in miRNA binding. For instance, in the FADS2 mRNA/mir331-3p pair, the G-quadruplex at the 3’UTR prevented the binding of the miRNA [164]. 3’UTRs most often contain alternative polyadenylation signals which can alter the regulation, stability and localization [178] (Fig. 4c). The G-quadruplexes at the 3’UTR of two genes, *LRP5* and *FXR1*, were tested with luciferase assay experiments and were found to increase the efficiency of alternative polyadenylation site usage and act as *cis*-regulatory elements that alter miRNA regulation [167] (Fig. 4d). In the lncRNA MALAT1, three G-quadruplexes in its 3’ end form stable RNA structures that can interact with proteins such as nucleolin and nucleophosmin in HeLa cells, and the G-quadruplexes are crucial for the localization of these proteins to nuclear speckles [179] (Fig. 4b, d).

Interestingly, within 100 base pairs downstream of the transcription end site there is a depletion of G-quadruplexes at the template strand as well as a profound enrichment at the non-template strand. Since the downstream enrichment is pronounced when there are neighboring genes in close proximity, it has been proposed that G-quadruplexes have a role in transcription termination [177]. In p53, a RNA G-quadruplex downstream of the gene controls pre-mRNA 3'-end processing regulation and is involved in the dynamic response to DNA damage [180]. The functional relevance of G-quadruplexes in 3’UTRs is also evidenced by the fact that they are selectively constrained and enriched for eQTLs, RBP sites and disease-associated variants. In a study of 150 RBPs, 15 RBPs were found to bind more often than expected by chance at G-quadruplex sites [181] (Fig. 4d, e).

R-loops are important for transcription termination, and they are over-represented at gene terminal regions, with a 3-fold enrichment [48]. The site of transcription termination is a major site of RNAPII pausing [182], and the R-loop formation enables the pausing and efficient transcription termination (Fig. 4d). Senataxin-deficient cells display transcription initiation, elongation and termination defects and increased RNAPII density downstream of the poly(A) signal. In particular, Senataxin resolves R-loop structures, enabling XRN2-mediated 3’ transcript degradation and RNAPII termination [183]. BRCA1 mediates the recruitment of Senataxin at R-loop sites, and in its absence there is increased mutagenesis at those sites [184]. Therefore, R-loops are needed for efficient transcription termination, but once formed they need to be resolved by Senataxin and BRCA1.

Hairpin structures at the 3’UTR of transcripts can modulate expression, mRNA stability and localization. They can also act as responsive elements to environment changes [185], and can conceal or expose cis-regulatory elements [165, 186] (Fig. 4e, f). For instance, the iron-responsive element is a hairpin structure found in 5’UTRs and 3’UTRs, which interacts with two iron regulatory proteins, IRP1 and IRP2 for cellular iron homeostasis [187]. Another example is the perinuclear localization of c-myc mRNA, which is controlled by a 3’UTR hairpin structure [188]. The constitutive decay element forms a hairpin at the 3’UTR of TNF-alpha [189], which Roquin and Roquin2 proteins can bind to promote mRNA decay. In addition, Roquin binds at the 3’UTR at hairpin RNA structures to mediate mRNA deadenylation [172]. Recent MPRA work, in which the biophysical properties of the hairpin of the constitutive decay element were altered, provided insights into the hairpin’s role in transcript levels and degradation rate [176, 190].

## Non-canonical secondary structures in disease

Mutations in the human genome are not distributed homogeneously. Sequences that are predisposed to secondary structure formation are mutational hotspots and their instability has been associated with the development of several diseases, including multiple neurological disorders [191, 192] and cancer [193, 194]. Therefore, advances in our understanding of the function of these sequences could have important implications in understanding cancer development, improve the etiology of other diseases and facilitate modeling evolution with higher precision.

Longer inverted repeats are more prone to mutagenesis than their shorter counterparts [195]. Negative supercoiling during transcription aids the formation of cruciforms and hairpins which in turn impact transcription and transcription factor binding [196–198]. At promoters, recurrent mutagenesis at inverted repeats has been observed across cancer types [199–201], which has been attributed to APOBEC off-target mutagenesis [201]. However, it remains unclear whether these recurrent mutations have a role in tumor progression [194]. Analysis of an inverted repeat at the *PLEKHS1* promoter that is recurrently mutated across disparate cancer types did not find reproducible evidence for changes in its expression levels [202]. This result contrasts with the findings for the TERT promoter G-quadruplex, which coincides with driver mutations.

Developmental genes and oncogenes [203], including *CKIT*, *KRAS*, C*MYC* and *BCL2*, are enriched for the presence of G-quadruplexes in their promoters. In particular, the promoter of the oncogene *CMYC* was one of the first where a role for G-quadruplexes in expression modulation was demonstrated. A pioneering study showed that both mutations that disrupt the structure formation and a stabilizing small molecule compound substantially alter expression levels in opposite directions [4]. Similarly, the *BCL2* promoter contains a G-quadruplex, whose stabilization with quindoline derivatives results in significantly decreased expression, while mutagenesis that disrupts formation of the structure increases expression [204]. Interestingly, the *TERT* promoter harbors the most frequent recurrently mutated sites across non-coding regions found in multiple cancer types [200, 202], and in ~90% of human cancers TERT expression is upregulated [205]. It has been shown that a G-quadruplex structure can form in the commonly mutated region of the TERT promoter, and stabilization by specific chemical compounds [206–208], leads to the down-regulation of TERT expression, directly implicating mutations of the G-quadruplex locus in carcinogenesis.

Inefficient repair of tandem repeats often leads to repeat expansions or contractions. There are over one million tandem repeats in the human genome, many of which are polymorphic, and their expansion is causal for many disorders [16, 209, 210] such as Huntington disease, spinocerebellar ataxias, Friedreich ataxia and Fragile X syndrome [16, 209, 210]. There is growing evidence that persistent R-loop formation can result in genomic instability [211] and R-loops are implicated in a number of diseases including cancers, autoimmune and neurological disorders [15, 43, 212]. The gene fusion of EWS-FLI or SS18-SSX in Erwin sarcoma has been shown to cause R-loop accumulation and increased replication stress [213]. In cells derived from Friedreich ataxia patients there is an accumulation of R-loops at the expanded GAA repeats of *FXN* gene which causes transcriptional repression [214], while introduction of anti-GAA duplex RNAs interferes with R-loop formation and restores FXN protein levels [215]. In spinal muscular atrophy, Senataxin-deficiency results in accumulation of R-loops, while its over-expression reverses this effect and rescues neurodegeneration [216, 217]. In Wiskott-Aldrich syndrome, which is due to a mutation in Wiskott-Aldrich syndrome protein, there is an accumulation of R-loops leading to genomic instability [218]. In Aicardi-Goutières syndrome, an excess of R-loops has been observed, especially at DNA hypomethylated sites [219].

## Technological advances and prospects

Advances in genome-wide and transcriptome-wide structure inference and visualization methods have allowed for quantification of the abundance and topological characteristics of multiple DNA and RNA structures. While rapid progress has been made recently, we believe that we are only starting to decipher the regulatory roles of secondary structures. Importantly, many recently developed technologies have not been implemented in this field.

One example of such a technology is single cell profiling methods which have the potential to identify relevant differences between cell types. Although it has been shown that G-quadruplex structure formation can be cell-type specific and is associated with higher expression levels and open chromatin [105], the degree to which non-B DNA and RNA structure formation is influenced by the tissue and cell type remains largely unstudied [220]. Single cell technologies could make it possible to apply genome wide assays or small molecules to individual cells to investigate the role of non-B DNA motifs across cell types.

Long-read sequencing technologies are required to map highly repetitive regions of the genome and the transcriptome, and a recent study provided evidence that centromeres are highly enriched in non-B DNA sequences [221]. Moreover, long microsatellite and minisatellite sequences are also routinely excluded from analyses due to prohibiting error rates and mapping problems [222]. Estimation of the full implication of short tandem repeat variation has been limited by sequencing technologies, but with long-read sequencing their contribution will become better understood.

High-throughput experiments enable the systematic investigation of thousands of sequences in a single experiment. Multiple technologies have been developed including those based on synthetic library designs such as massively parallel reporter assays and those based on genome-fragmentation approaches such as STARR-seq [223, 224]. They have provided valuable insights regarding the roles of non-canonical secondary structures in promoters [117], 5’UTRs [225] and 3’UTRs [176].

The field has benefited from a plethora of G-quadruplex ligands, e.g PDS, cPDS, BRACO19, Phen-DC3, L2H2-6OTD, L1H1-7OTD, TMPyP4, which differ in their binding preference for DNA or RNA G-quadruplexes and can shift the equilibrium between folded and unfolded states. The modulation of specific DNA and RNA secondary structures with high specificity could allow for treatments of numerous disorders, including cancer and neurological disorders [226]. For example, Quarfloxin, which interacts with G-quadruplexes [227], reached Phase II trials for several cancer types. Unfortunately, Phase III trials are currently not proceeding due to side effects [228, 229]. CX-5461 is a G-quadruplex ligand that is currently part of a phase I clinical trial (NCT02719977) due to its cytotoxicity to cancer cells, e.g. those that are BRCA1-deficient or BRCA2-deficient [230], and it has been shown to exert its effects due to induction of G-quadruplex formation and topoisomerase II poisoning [231]. Finally, there is a number of pre-clinical studies that suggest that G-quadruplexes could have therapeutic potential, an example being the G-quadruplex ligand CM03, which binds to G-quadruplexes and down-regulates the expression of multiple genes that are involved in pancreatic ductal adenocarcinoma survival, metastasis and drug resistance [232].

G-quadruplexes can be characterized across the genome [56] and the transcriptome [57] using methods based on high-throughput sequencing. Similarly, methods have been developed to identify R-loops genome-wide [70, 233]. The combination of permanganate footprinting with high-throughput sequencing has enabled the genome-wide detection of single-stranded DNA and the deduction of non-B DNA structures [234]. However, such methods are currently lacking for other non-B DNA structures, e.g. hairpins and slipped structures. The development of novel antibodies and probe technologies could enable the estimation of the frequency and localization of each type of non-B DNA structure globally and could provide insights into how they can switch between their unfolded and folded states.

The cellular mechanisms mediating the stabilization of DNA and RNA secondary structures and those that resolve them, e.g. RBPs, remain incompletely understood. In addition, the effect of interrupting the function of these mechanisms and the relevance to disease progression is unclear. Recent development of high throughput screens coupled with short hairpin RNAs (shRNAs) or CRISPR-based technologies have enabled systematic interrogation of the roles of diverse proteins, such as transcription factors, RBPs, helicases, and topoisomerases. Mutational analysis with CRISPR-Cas9 could also be used to study the effects of non-B DNA motif disruption *in vivo*, while variants of the technology without endonuclease activity could also be used to elucidate their functions.

### Concluding Remarks

High-throughput technologies enable the systematic investigation of non-canonical secondary structures as well as the design of experiments to quantify their contribution in the regulation of gene expression and to directly testing their mechanisms of action. The discovery of new methods to dynamically identify non-B DNA and RNA structures is gradually revealing their widespread and diverse contributions in gene regulation. However, it remains difficult to capture their dynamic changes across cellular conditions and their interplay with proteins. We believe that the implementation of novel technologies will enable breakthrough discoveries for their roles with important implications in our understanding of gene regulation. Crucially, a better understanding of the mechanisms through which secondary structures impact gene expression will allow for the development of novel therapeutic strategies for a wide range of diseases, including cancer and neurodegenerative disorders.

## Supplementary Information

**Additional file 1.** Review history.

## Data Availability

Not applicable as there are no analyses of primary data included in the manuscript.
