# Author Correction: High-throughput techniques enable advances in the roles of DNA and RNA secondary structures in transcriptional and post-transcriptional gene regulation

**DOI:** 10.1186/s13059-022-02733-8

**Published:** 2022-07-27

**Authors:** Ilias Georgakopoulos-Soares, Candace S. Y. Chan, Nadav Ahituv, Martin Hemberg

**Affiliations:** 1 Department of Bioengineering and Therapeutic Sciences, University of California San Francisco, San Francisco, California USA; 2 Institute for Human Genetics, University of California San Francisco, San Francisco, California USA; 3 Evergrande Center for Immunologic Diseases, Harvard Medical School and Brigham and Women’s Hospital, Boston, MA USA

**Correction: Genome Biol 23, 159 (2022)**

**https://doi.org/10.1186/s13059-022-02727-6**

Following publication of the original article [1], it was noticed that the legends for Fig. 1 and Fig. 2 had been switched. The correct legends are given below.

**Fig.** 1 Schematics of DNA and RNA structures. **A** The canonical right handed double helix, also known as B DNA secondary structure. **B** Z-DNA forms a left-handed double helix. **C** G-quadruplexes are formed by the stacking of multiple G-quartets held together by Hoogsteen hydrogen bonds (top). Four guanines establish hydrogen bonds with each other to form a G-quartet (bottom). Hoogsteen hydrogen bonds are highlighted in blue. The monovalent cation that can stabilize the G-quadruplex structure is marked with M. **D** Hairpins are formed at inverted repeats, in which the stem base pairs hybridize with hydrogen bonds, while the loop remains single-stranded. **E** Slipped-strand mispairing at tandem repeats results in slipped structure formation. **F** Depiction of a homopurine-homopyrimidine sequence with mirror symmetry. H-DNA is a triple helix secondary structure where the third strand hybridizes with Hoogsteen hydrogen bonds with the duplex DNA, while the fourth strand remains single stranded. **G** R-loops are formed co-transcriptionally at the template strand. The nascent RNA produced by the RNA-polymerase hybridizes with the template strand to form an R-loop structure, while the non-template strand remains single-stranded

**Fig.** 2 Schematic overview of Non-B DNA enrichment relative to gene features. Higher density of non-B DNA structures is observed at promoter regions, 5’UTRs, regions flanking splice sites and at the 3’UTR. Formation of secondary structures is also facilitated by negative supercoiling and at actively transcribed regions relative to the direction of the transcribing RNA polymerase [2, 3]
