# Supplementary material for: High-throughput techniques enable advances in the roles of DNA and RNA secondary structures in transcriptional and post-transcriptional gene regulation
Source: Genome Biol. 2022 Jul 18;23:159. doi: 10.1186/s13059-022-02727-6 (PMC9290270; doi:10.1186/s13059-022-02727-6)
Supplement: Supplementary file 1 — Additional file 1. Review history. [file 13059_2022_2727_MOESM1_ESM.docx]

**1^st^ round**

**Reviewer 1**

**Is the topic of the article timely and of interest to a wide range of readers?**

Yes

**Is the article well written and presented in a logical way?**

Yes

**Do the authors cover the relevant literature in an accurate and balanced way?**

Yes

**Do the authors provide a useful synthesis of the topic?**

Yes

**Do the authors provide insightful discussion of the future directions for the field?**

Yes: As indicated above, this review is timely and well written. There is some discussion for future prospects, but it can be elaborated a little more. This section is a little short.

**Do any tables, figures or boxes that are present enhance the manuscript?**

Yes

**Would the manuscript benefit from any additional tables, figures or boxes?**

No: The manuscript does not need more figures

**Comments:**

A couple of points could be clarified better. Eg where the authors discuss the differences between G4 seq and G4 ChIP in terms of variability in peak number (pg6, ln 6), they can include that perhaps the antibodes may detect only some forms of G4?

In the same page where Rnase H methods are mentioned, the authors should cite R-CHIP. This was the first RNase H method described (Chen et al Molecular Cell).

In Pg 9 - Lines32-36. This is a little confusing. Are the authors refering to triplex structures or R-loops, which are three stranded structures, but different from triplexes.

**Reviewer 2**

**Is the topic of the article timely and of interest to a wide range of readers?**

Yes

**Is the article well written and presented in a logical way?**

No: There is no links between the DNA secondary structure and RNA secondary structure. In particular, the part of RNA structure is more related to the post-transcriptional regulation rather than transcriptional regulation. The session of non-canonical secondary structure in disease is also quite closely related to the other sessions.

**Do the authors cover the relevant literature in an accurate and balanced way?**

No: The references used are quite old.

**Do the authors provide a useful synthesis of the topic?**

No: The sub-titles are not coherent.

**Do the authors provide insightful discussion of the future directions for the field?**

No

**Do any tables, figures or boxes that are present enhance the manuscript?**

No: The figures are too simple to illustrate the information clearly

**Would the manuscript benefit from any additional tables, figures or boxes?**

No: The authors need to think about how to present the figures.

**Comments**

The topic itself is very interesting. However, there is no links between the DNA secondary structure and RNA secondary structure. In particular, the part of RNA structure is more related to the post-transcriptional regulation rather than transcriptional regulation. The session of non-canonical secondary structure in disease is also quite closely related to the other sessions. The whole story line is quite unclear. The detailed comments are as follows:

1. In the session of non-canonical DNA and RNA secondary structure, most references related to DNA structures such as Z-DNA are very old. Only the development on studying DNA GQS is recent. However, the authors did not provide a detailed overview on it. For instance, the advantages and disadvantages of these high throughput methods are not clear.

2. In the session of "Secondary structures are involved in transcriptional regulation at promoters", the author focused on the study on in silico predicted DNA secondary structure that are associated with PolII pausing. This study is more general for PolII pausing rather than the transcriptional regulation at promoters. The main message in this session is not clear. All the references were collected without a clear line.

3. The followed session of "transcription termination and secondary structure" did not connect with the session of "Secondary structures are involved in transcriptional regulation at promoters" since the authors directly jumped from the DNA structure to RNA structure. The polyadenylation, splicing and RNA degradation are more related to the post-transcriptional regulations rather than transcription termination.

4. Again, in the session of "non-canonical secondary structure in disease", the authors started with mutations in human disease and then jumped to the potential links between GQS/R-loop and human disease. The used references are not strongly evident in supporting this session.

**Reviewer 3**

**Is the topic of the article timely and of interest to a wide range of readers?**

Yes

**Is the article well written and presented in a logical way?**

Yes

**Do the authors cover the relevant literature in an accurate and balanced way?**

Yes

**Do the authors provide a useful synthesis of the topic?**

Yes

**Do the authors provide insightful discussion of the future directions for the field?**

Yes: Mainly in the concluding remarks

**Do any tables, figures or boxes that are present enhance the manuscript?**

Yes

**Would the manuscript benefit from any additional tables, figures or boxes?**

No: Those present are sufficient

**Authors’ response**

Reviewer #1: A couple of points could be clarified better. Eg where the authors discuss the
differences between G4 seq and G4 ChIP in terms of variability in peak number (pg6, ln 6), they can include that perhaps the antibodes may detect only some forms of G4?
We thank the reviewer for the comment and we agree with the suggestion. We have now revised this paragraph to include this possibility and clarify better we provide below the two changes:
“This discrepancy could be the result of rapid resolution of G-quadruplexes by helicases in the cellular environment [56], it could reflect differences in the genomic locality, associated with epigenetic changes and chromatin accessibility [57] and could be biased by the chemical perturbation with K+ or Pyridostatin (PDS) in G4-seq experiments. In addition, in ChIP-seq this discrepancy could also be explained by the antibody being able to recognize only certain G-quadruplexes. Antibody-associated differences are indeed observed between BG4 and D1 antibodies [1,58].”
We also added the following to better clarify PDS and K+ and the limitations of these high-throughput methods:
“Large variability in the number of peaks has also been observed in G4-seq experiments where the G-quadruplex stabilization method seems to influence the results with substantial differences in PDS and K+ treatments. PDS is a small molecule compound that has been shown to bind to G-quadruplexes, leading to their stabilization [63]. Similarly, K+ ions interact with G-quadruplexes and stabilize them. High-throughput G-quadruplex detection methods identify sites that do not conform to the consensus G-quadruplex motif. However, these high-throughput methods entail certain limitations. These include the inability to discern the G-quadruplex forming potential between different cell types or to measure temporal effects; they do not provide information about the kinetics and thermal stability of G-quadruplexes at individual loci.”

In the same page where Rnase H methods are mentioned, the authors should cite R-CHIP. This was the first RNase H method described (Chen et al Molecular Cell).
We thank the reviewer for pointing this out. We have added:
“R-ChIP was the first method that mapped genome-wide R-loops using catalytically dead RNaseH coupled with by amplification of immunoprecipitated DNA [72].”

In Pg 9 - Lines32-36. This is a little confusing. Are the authors refering to triplex structures or R-loops, which are three stranded structures, but different from triplexes.

We thank the reviewer for pointing this out it was indeed not completely clear, we have modified the text:
“Triple helix structures (single-strand RNA hybridizing to double-stranded DNA with Hoogsteen bonds) have been shown to contribute towards genome organization [84], and some of the most thoroughly studied lncRNAs, e.g. HOTAIR, MEG3 and PARTICLE, form triple stranded DNA-RNA hybrids to perform epigenetic modifications and to regulate gene expression [140–142].”

Reviewer #2: The topic itself is very interesting. However, there is no links between the DNA secondary structure and RNA secondary structure. In particular, the part of RNA structure is more related to the post-transcriptional regulation rather than transcriptional regulation. The
session of non-canonical secondary structure in disease is also quite closely related to the other sessions. The whole story line is quite unclear. The detailed comments are as follows:
We thank the reviewer for their thorough reading of the manuscript and the constructive comments. We have changed the title to indicate that we are covering both transcriptional and post-transcriptional regulation. We regret the lack of clarity of the narrative, and we hope that the new version is clearer.

1. In the session of non-canonical DNA and RNA secondary structure, most references related to DNA structures such as Z-DNA are very old. Only the development on studying DNA GQS is recent. However, the authors did not provide a detailed overview on it. For instance, the advantages and disadvantages of these high throughput methods are not clear.
We thank the reviewer for pointing this out and agree that G-quadruplex and R-loop are the secondary structures with the most active research. We have expanded the section on G-quadruplexes to describe the advantages and disadvantages of these recently developed high-throughput methods and have improved this section which we also provide below:
”Genome-wide maps of sequences that can form G-quadruplexes in vitro under favourable conditions have been generated using a modified sequencing method that stalls at G-quadruplexes [29,56]. This technique has been termed G4-seq. Similarly, rG4-seq, which is a variant of G4-seq, has enabled transcriptome-wide identification of RNA G-quadruplexes [57]. Recently, a novel method called G4-miner was used for genome-wide profiling of
G-quadruplexes from standard whole-genome sequencing based on deviations in sequencing quality, with drop in sequencing quality at G-quadruplex structures [58]. Antibodies with high affinity for G-quadruplexes have been used for G4 ChIP-seq experiments to demonstrate genome-wide G-quadruplex structure formation in human cells [1,59]. Crucially, the number of loci discovered forming G-quadruplexes by ChIP-seq in vivo is ~10,000 while G4-seq in vitro identifies ~700,000 peaks [29,56,59]. This discrepancy could be the result of rapid resolution of G-quadruplexes by helicases in the cellular environment [60], it could reflect differences in the genomic locality, associated with epigenetic changes and chromatin accessibility [61] and could be biased by the chemical perturbation with K+ or Pyridostatin (PDS) in G4-seq experiments. In addition, in ChIP-seq this discrepancy could also be explained by the antibody being able to recognize only certain G-quadruplexes. Antibody-associated differences are indeed observed between BG4 and D1 antibodies [1,62]. Large variability in the number of peaks has also been observed in G4-seq experiments where the G-quadruplex stabilization method seems to influence the results with substantial differences in PDS and K+ treatments. PDS is a small molecule compound that has been shown to bind to G-quadruplexes, leading to their stabilization [63]. Similarly, K+ ions interact with G-quadruplexes and stabilize them. High-throughput G-quadruplex detection methods identify sites that do not conform to the consensus G-quadruplex motif. However, these high-throughput methods entail certain limitations. These include the inability to discern the G-quadruplex forming potential between different cell types or to measure temporal effects; they do not provide information about the kinetics and thermal stability of G-quadruplexes at individual loci. More recently, single molecule G-quadruplex tracking in living cells, using a G-quadruplex specific fluorescent probe, has indicated dynamic fluctuations between folded and unfolded states [64].”

2. In the session of "Secondary structures are involved in transcriptional regulation at promoters", the author focused on the study on in silico predicted DNA secondary structure that are associated with PolII pausing. This study is more general for PolII pausing rather than the transcriptional regulation at promoters. The main message in this session is not clear. All the references were collected without a clear line.
We thank the reviewer for their comment. In that section we describe a plethora of mechanisms by which secondary structures regulate promoter activity structured by evidence for each secondary structure. The reviewer is correct in that we do not provide enough discussion in RNAPII pausing specifically at promoters, which only briefly mention. We cite a recently published work by Eddy et al. where they demonstrate an association between G-quadruplex motifs and promoter RNAPII pausing (PMID: 21371997). The findings are targeted at promoters, at sites of RNAPII pausing. We also cite the work of Szlachta et al. 2018, which addresses RNAPll promoter-proximal pausing and the association with DNA secondary structures (PMID:30001206). This study used ChIP-seq, GRO-seq, NET-seq, and mNET-seq data to arrive at these conclusions specifically at promoter-proximal pausing.
We also would like to highlight the paper of Chen et al. 2017, in which they provide evidence using transcription perturbation experiments for a strong link between R-loop induction with RNAPII pausing at gene promoters specifically (PMID: 29104020). In addition, the paper by Zhang et al. 2017 (PMID: 28649985) show that R-loops accumulate at the 5′ end of genes with promoter-proximal RNAPII pausing when BRCA1 is not functional, which has been previously shown to resolve R-loops . Finally, other studies have provided experimental and bioinformatic evidence for transcription blockade due to R-loop formation at promoter-proximal regions (PMID: 23275544, PMID: 26275623).
We have now expanded this section to reflect these studies more thoroughly which we provide below:
“Roles for R-loops in long non-coding RNA (lncRNA) transcription have also been shown, in
which R-loop formation can induce lncRNA generation which in turn act as transcriptional inducers [134,135]. Specifically, at promoters R-loop formation can generate antisense lncRNAs, and their removal results in the inhibition of antisense lncRNAs [135]. R-loops have been found to be important for lncRNA function, for example the HOTTIP lncRNA recruits CTCF and cohesin, interacts with R-loop associated proteins and induces the formation of R-loops at TAD boundaries, which in turn regulates and reinforces those boundaries [100]. In Arabidopsis thaliana, the lncRNA APOLO recognizes distal targets via R-loops to epigenetically silence them [136]. There is also accumulating evidence for roles of R-loops in promoter-proximal RNAPII pausing [72,137–139]. Transcription perturbation experiments have provided evidence for a strong link between R-loop induction and RNAPII pausing near transcription start sites [72].
Another line of evidence comes from a study which showed that BRCA1 can resolve R-loops at promoter-proximal RNAPII pausing sites. [139], while in BRCA1 mutant cells, R-loops
accumulate at the 5′ end of genes resulting in promoter-proximal RNAPII pausing [139].“

3. The followed session of "transcription termination and secondary structure" did not connect with the session of "Secondary structures are involved in transcriptional regulation at promoters" since the authors directly jumped from the DNA structure to RNA structure. The polyadenylation, splicing and RNA degradation are more related to the post-transcriptional regulations rather than transcription termination.
We thank the reviewer for the comment and agree the section title could be improved. We have now changed the title to:
“Secondary structures at the 3’UTR in transcriptional and post-transcriptional regulation”
Which reflects both the transcription termination effects and mechanisms as well as those at the post-transcriptional level.

4. Again, in the session of "non-canonical secondary structure in disease", the authors started with mutations in human disease and then jumped to the potential links between GQS/R-loop and human disease. The used references are not strongly evident in supporting this session.
We thank the reviewer for pointing this out. We have restructured this section, added additional references and provided more information about the link between non-canonical secondary structure and human diseases. In particular, we have also explained the paragraph on R-loops and human disease. We provide below that section:
“There is growing evidence that persistent R-loop formation can result in genomic instability
[211] and R-loops are implicated in a number of diseases including cancers, autoimmune and neurological disorders [14,43,212]. The gene fusion of EWS-FLI or SS18-SSX in Erwin sarcoma has been shown to cause R-loop accumulation and increased replication stress [213]. In cells derived from Friedreich ataxia patients there is an accumulation of R-loops at the expanded GAA repeats of FXN gene which causes transcriptional repression [214], while introduction of anti-GAA duplex RNAs interferes with R-loop formation and restores FXN protein levels [215]. In spinal muscular atrophy, Senataxin-deficiency results in accumulation of R-loops, while its over-expression reverses this effect and rescues neurodegeneration [216,217]. In Wiskott-Aldrich syndrome, which is due to a mutation in Wiskott-Aldrich syndrome protein, there is an accumulation of R-loops leading to genomic instability [218]. In Aicardi-Goutières syndrome, an excess of R-loops has been observed, especially at DNA hypomethylated sites [219].”

Reviewer #3: In this review the roles of non-canonical nucleic acid secondary structures in
transcription are presented.
The review is concise, clear and orderly.
We thank the reviewer for the overall positive assessment and the constructive inputs.

Main comments
Page 12 lines 48-58. Reference Georgakopoulos-Soares et al. 2021, which is not published yet in a peer-review journal, is mentioned to report that G-quadruplexes do not affect expression levels. However, many papers have now shown that G-quadruplexes are associated with increased levels of transcripts, as also mentioned in the previous paragraphs by the authors. How these two contrasting pieces of information coexist? This discrepancy has to be thoroughly discussed.
We apologize for this. The paper was published in Cell Genomics in March 2022, and we have now corrected this reference and added it in the bibliography. The paper finds the increased expression associated with G-quadruplexes is confounded by higher GC content, at least in massively parallel reporter assay experiments, and when adjusting for GC content
G-quadruplexes are no longer associated with increased expression. However it would be of
interest to investigate how treatment with G-quadruplex stabilizing molecules could affect these conclusions. We discuss these conclusions in the review which we provide below:
“A recent high-throughput massively parallel reporter assay (MPRA) study examined the contribution of promoter G-quadruplexes and reported a positive correlation between their presence and expression levels. However, after accounting for GC-content differences, G-quadruplexes were either not significantly associated with expression levels or were associated with reduced gene expression [117]. “

Page 18: quarfloxin is not the only G-quadruplex ligand that entered clinical trials. Please check and correct.
We have now expanded this section and we also provide it below::
“The modulation of specific DNA and RNA secondary structures with high specificity could allow for treatments of numerous disorders, including cancer and neurological disorders [226]. For example, Quarfloxin, which interacts with G-quadruplexes [227], reached Phase II trials for several cancer types. Unfortunately, Phase III trials are currently not proceeding due to side effects [228,229]. CX-5461 is a G-quadruplex ligand that is currently part of a phase I clinical trial (NCT02719977) due to its cytotoxicity to cancer cells, e.g. those that are BRCA1-deficient or BRCA2-deficient [230], and it has been shown to exert its effects due to induction of G-quadruplex formation and topoisomerase II poisoning [231]. Finally, there is a number of pre-clinical studies that suggest that G-quadruplexes could have therapeutic potential, an example being the G-quadruplex ligand CM03, which binds to G-quadruplexes and down-regulates the expression of multiple genes that are involved in pancreatic ductal adenocarcinoma survival, metastasis and drug resistance [232].”

No mention to the A form of DNA is present. Should not it also warrant a mentioning
We agree with the reviewer. We have now revised this to include A-DNA which we provide below:
“Double stranded DNA is composed of two helices held together by hydrogen bonds, and most often adopts the canonical right-handed double helical secondary structure, also known as B-DNA (Figure 2a), with 10.5 residues per turn [18]. Similarly to B-DNA, A-DNA is also
right-handed and double helical albeit with 11 residues per turn and forms more readily at GC-rich regions [19,20].”

Since the focus of the review is mainly on high-throughput techniques in cells, this aspect should be present in the title.
We thank the reviewer for their input. We have now revised the title to:
“High-throughput techniques enable advances in the roles of DNA and RNA secondary structures in transcriptional and post-transcriptional gene regulation”

Typos

In Figure 2 legend: typo in the word multiple
We thank the reviewer for pointing this out. We have now corrected this.

Page 9 line 35: revise the following sentence:
Recently, permanganate footprinting coupled was combined with genome-wide sequencing to identify transiently formed single stranded DNA regions [1],
We thank the reviewer, we have now corrected this sentence.

Figure 3: CTCF binding is mentioned in the legend to the figure, but it is not clear where the
pertinent figures (which panels?) are.
We thank the reviewer and apologize for this omission. We have now revised this figure to include more details including annotation of CTCF.

This reference is mentioned without the formatting used for all other references
(Georgakopoulos-Soares et al. 2021). Also, in the bibliography, the full reference is missing. If the paper is not published yet, it cannot be mentioned.
We apologize for this. The paper has now been through peer-review and we have now corrected this reference and added it in the bibliography.

Legend to figure 4: typo in "e" panel in the legend. Also, legend descriptions to panels e-g do not correspond to the figures.
We apologize for this. We have now corrected the legend and fixed the order.

References 1 and 53 are duplicates.
We thank the reviewer for pointing this out. We have now corrected this and removed the
duplicate reference.

Page 18: One example of such a technology is single cell profiling methods which have the
potential to identify relevant differences between cell types: is ref 153 the correct reference to be mentioned here? Please double check.
We agree with the reviewer and thank them for pointing this out. We have now removed this
reference as it was wrongly placed in that sentence
